# The Howiesons Poort lithic sequence of Klipdrift Shelter, southern Cape, South Africa

**DOI:** 10.1371/journal.pone.0206238

**Published:** 2018-11-07

**Authors:** Katja Douze, Anne Delagnes, Sarah Wurz, Christopher Stuart Henshilwood

**Affiliations:** 1 Department of Genetics and Evolution, Anthropology Unit, Archaeology and Population in Africa, University of Geneva, Geneva, Switzerland; 2 Centre National de la Recherche Scientifique, Unité Mixte de Recherche 5199, De la Préhistoire à l’Actuel: Culture, Environnement et Anthropologie, University of Bordeaux, Pessac, France; 3 School of Geography, Archaeology and Environmental Studies, University of the Witwatersrand, Johannesburg, South Africa; 4 Department of Archaeology, History, Cultural Studies and Religion, Centre for Early Sapiens Behaviour, University of Bergen, Bergen, Norway; 5 Evolutionary Studies Institute, University of the Witwatersrand, Johannesburg, South Africa; Max Planck Institute for the Science of Human History, GERMANY

## Abstract

Howiesons Poort (HP) sites, over the past decades, have provided exceptional access to anthropogenic remains that are enhancing our understanding of early modern human behaviour during the Middle Stone Age in southern Africa. Here, we analyse the technological and typological trends in the lithic record that form part of these behaviours, based on the HP sequence recently excavated at Klipdrift Shelter, located on the southern Cape coast of South Africa. This study contributes to enhance knowledge on the mechanisms of changes that occurred during the transition to the post-HP. Despite patterns of continuity observed, notably for core reduction methods, the seven successive lithic assemblages show significant changes in the typological characteristics and raw material selection but also in the relative importance of blade production over time. However, these changes are not necessarily synchronic and occur either as gradual processes or as abrupt technological shifts. Consequently, we cross-examine the association between the lithic phasing and other anthropogenic remains within the HP sequence at Klipdrift Shelter. We explore the implications of these patterns of changes in terms of cultural behaviours and population dynamics during the HP and we highlight the relationship between the different phases of the HP sequence at Klipdrift Shelter and those from other South African HP sites.

## Introduction

The Howiesons Poort (HP) is one of the most intensively discussed periods of the Late Pleistocene Middle Stone Age (MSA) in southern Africa. This is in part due to outstanding complex material culture, such as geometrically engraved ostrich eggshells and worked ochre pieces [1–7], found in association with blade-based industries, from which blanks are backed and hafted as possible arrows heads [8–10] and barbs [11], or used as cutting tools [12].

The HP is often considered as an exceptional and short-lived cultural unit within the MSA (see [13] with techno-cultural characteristics that are widespread across southern Africa (see e.g. [14–17]) during MIS 4 to 3. Recent studies have focused on a more precise definition of the HP in terms of chronology but also on its cultural characteristics over time and their regional variability. The excavations at Diepkloof, have shown that the HP likely occupies a more extended chronological period within the MSA than previously thought, with an emergence of an Early HP phase at ca. 109 ka (e.g. [18–19]) at this site although these ages have been queried [20]. The vision of a HP being a long-lasting system has also been suggested recently by other researchers [17]. Additionally, it is well established that regional variability exists across South Africa in specific tool types comprising the general HP lithic repertoire (e.g. [21]). Finally, a degree of convergence of HP technologies with regards to older or younger MSA technologies is also further explored. The best example is the focus on bifacial points as being part of the HP toolkits [13,19,22,23]. Although already recognized within the HP [24–33], such tool types were traditionally considered cultural markers of the preceding Still Bay industries (e.g. [14,34]).

While it is undoubtedly a distinct period within the MSA, the details of the HP still need to be better understood, as it seems to be characterized by important internal changes. Previous work on long HP sequences in southern Africa [1,5,13,15,19,32,35–37] has shown that tool manufacturing, raw material selection and blade *versus* flake production are the main markers of change through time. These changes have led to the identification of a number of phases within the HP (e.g. [19,35,36]). However, the investigation of the driving factors and mechanisms for transitions *within* the HP is usually overshadowed by the question of the transition to the post-HP, which is seen as a major turnover in lithic strategies and lifestyles (e.g. [13,23,36,38–41]).

Here we investigate seven successive HP layers at Klipdrift Shelter (KDS), located on the southern Cape coast of South Africa [5] and explore the nature and significance of changes occurring within these layers over time. Our preliminary analysis of the lithic assemblages have shown that there are two main HP phases within the KDS sequence, as well as a transitional HP phase possibly followed by a Post-HP layer [5]. We further develop our results here and discuss the process of behavioral changes over time. We address in particular the role of different proxies within the lithic system as well as patterns of change of the other material culture recovered at KDS to investigate the evolutionary dynamics within the HP and the factors involved in the transition to the post-HP.

## Material and methods

### Site background

**Fig 1:**
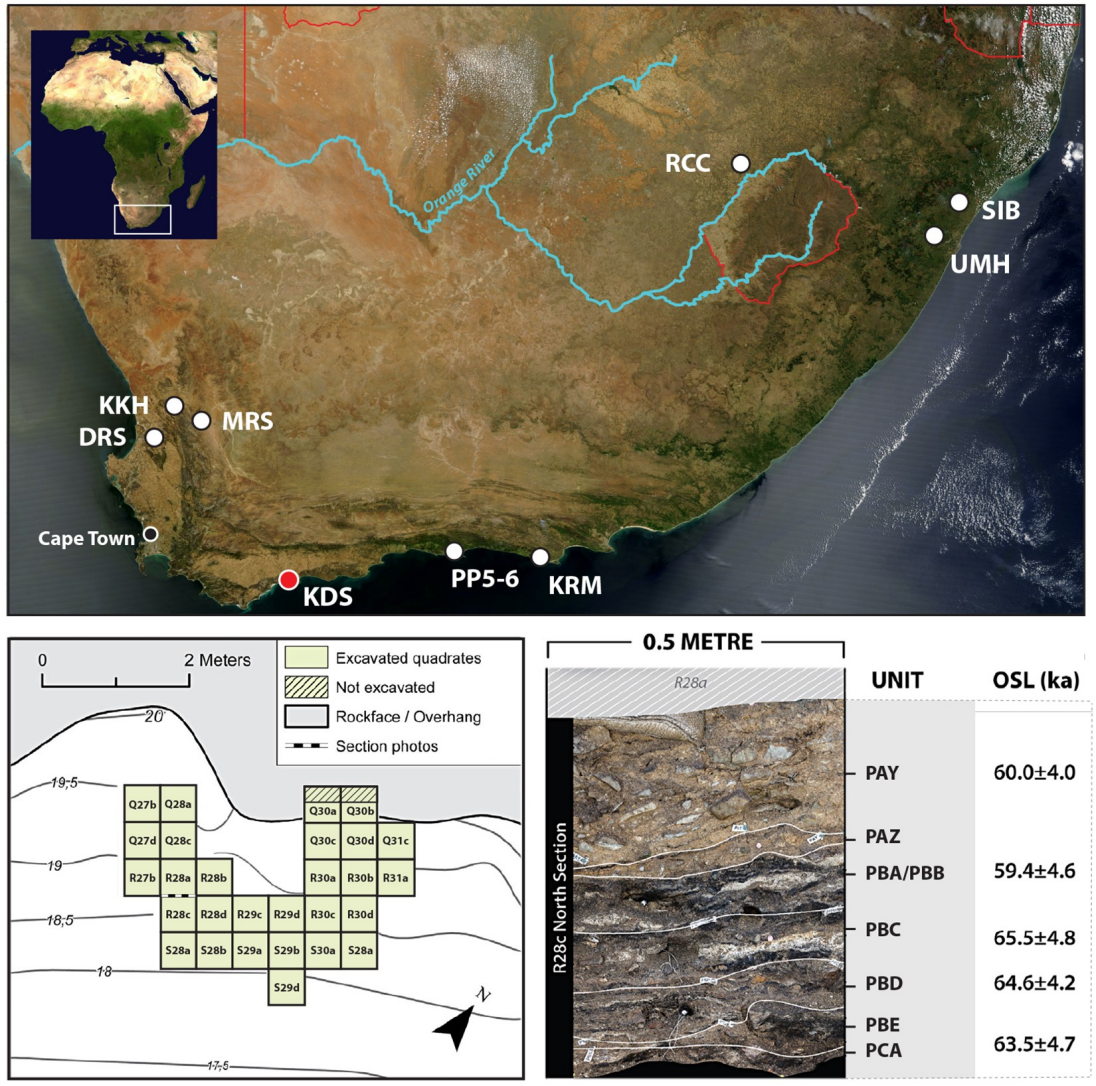
Location of KDS and other sites mentioned in the text. KKH: Klein Kliphuis; DRS: Diepkloof Rock Shelter; MRS: Mertenhof Shelter; KDS: Klipdrift Shelter; PP5-6: Pinnacle Point Site 5–6; KRM: Klasies River Mouth; RCC: Rose Cottage Cave; UMH: Umhlatuzana; SIB: Sibudu Cave. Satellite images modified after NASA, MODIS Rapid Response Team image, public domain. Location of excavated quadrates within KDS and simplified illustration of KDS HP stratigraphy (modified after [5]).

KDS is the eastern most shelter of the Klipdrift Complex (Fig 1) located on a steep slope angling towards the Indian Ocean seashore, in De Hoop Nature Reserve, southern Cape. It is formed from a fault breccia of the Table Mountain Group sandstones in the coastal cliff and is now 7 meters deep. KDS was first excavated in 2011 with three subsequent seasons in 2012, 2013 and 2018 to depths of 0.3 to 1 meter depending on the excavation area. Seven HP layers, from PCA at the base to PAY at the top, were recovered in the lower part of a sequence comprising a total of 23 layers and lenses [5]. Layers that exhibited a close contextual relationship during excavation, based on their lithostatigraphic features, were given names that share the two first letters (eg. PBC and PBE). In general terms, the layers included in PC and PB show alternating black, grey, white, and sometimes red-yellowish lenses of greasy compact texture and sometimes looser crumbly matrixes. The PAZ and PAY layers are distinguished by a sandy yellow to brown matrix with intermittent white ashes, in addition to large roofspalls in PAY (Fig 1). Based on these discrete lithostratigraphic features, we consider these layers independently for our study. Several Optically Stimulated Luminescence (OSL) dates were obtained on the HP sequence, indicating ages of between 65.5±4.8 ka and 59.4±4.6 ka [5] and arebroadly consistent with OSL dates obtained for other HP sequences. The site has provided a large lithic collection, numerous faunal remains [42–44] and shellfish collections as well as ochre and ostrich eggshell, some which are worked or engraved [5]. Additionally, Delagnes et al. [45] described how the heat-treatment of silcrete at KDS impacted the entire *chaîne opératoire* of blade production in layer PBD.

### Assemblages under study

Our analysis is based on all of the HP layers, named (from the lowest level): PCA, PBE, PBD, PBC, PBA/PBB, PAZ, PAY. A total of 6,837 lithics were recorded individually, in addition to 4,837 chunks and pebbles which are not further discussed here. The chunks are mainly from quartzite and are potentially products of roof spalling [see 5], while small pebbles (<3cm) with no visible transformation, come from the beach below. The studied sample is composed of all lithics ≥ 2cm and all retouched tools and bladelets <2cm.

Table 1 provides the average density of lithics under study for a quadrate (¼ m² or 50 x 50 cm) in addition to their total amount per layer. Although there are differences in average densities, layer PBA/PBB being by far the densest and PAZ the least dense, we consider the total sample available for each layer sufficient for our analyses.

**Table 1:** General counts and composition of lithic material studied at KDS (exclusive of unretouched chunks and pebbles) and an indication of the density of lithics per layer.

Layer	Number of quadrates studied	Average density per quadrate n=	Total lithics n=	Cores n=	Flakes n=	Blades n=	Other n=
PAY	7	107	749	25	527	197	0
PAZ	6	87	523	19	399	103	2
PBA/PBB	5	309	1544	47	1093	403	1
PBC	4	141	645	30	466	148	1
PBD	13	141	1838	54	966	813	5
PBE	4	197.5	790	12	373	404	1
PCA	5	150	748	16	427	305	0

The total lithic counts includes all lithics ≥ 2 cm as well as complete small blades and retouched tool fragments <2 cm. The category ‘Other’ represents retouched chunks or indeterminate lithic types.

We use the term blade for all products that fit the size ratio of length ≥ 2 x width, regardless their maximal length, as it follows a unimodal distribution, as observed at Rose Cottage Cave [17]. The blade category thus includes from very small to large blades, whole or broken. All other products (including laminar flakes, flakes with laminar negative scars etc.) are integrated to the flake class of Table 1.

## Results

### Raw material selection

**Fig 2:**
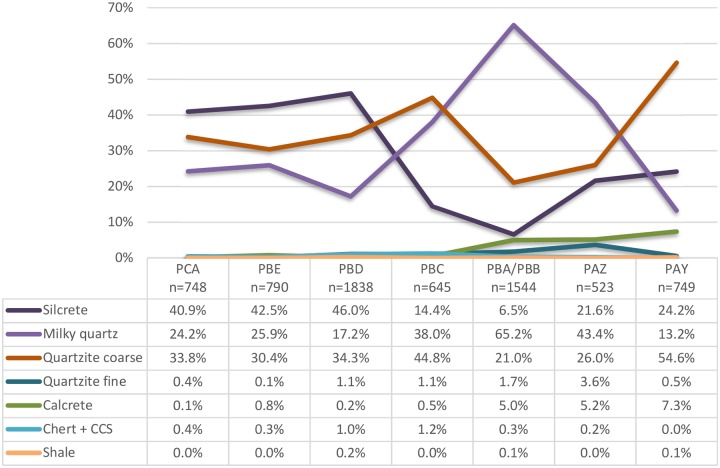
Relative proportions of main raw material types per layer at KDS (lithics ≥2cm).

Different categories of raw material are represented in the HP sequence of KDS for pieces >2cm, among which silcrete (29%), coarse quartzite (33%) and milky quartz (33%) are by far the most frequent when all the layers are considered together. However, important changes in their respective proportions occur through the sequence, reflecting significant changes in raw material selection over time (Fig 2).

The milky quartz at KDS is highly homogeneous in structure. Quartz blades and cores with remnant cortex (3.4%) show either natural surfaces (plain angular or irregular) or pebble surfaces in similar proportions. Quartz is currently available in the direct vicinity of the shelter, on the beach beneath the site and in the Cape Supergroup sequence near the site. Crystal quartz (ie. transparent), represented in low proportions (Fig 2), derive from variations found within the milky quartz structures, that are present in and near the site.

Quartzite is mostly coarse and corresponds better to the quartzite type available in the shelter walls than to the fine grained quartzite cobbles available on the beach beneath the site. The rare coarse quartzite cores and blades bearing cortical surfaces (1.2%) confirm this observation since they show planar natural fractures rather than cobble waterworn cortex. Cobbles have been exclusively selected for blade production as all cores and blades are in fine quartzite, sometimes associated with remnant cobble cortex.

The utilised silcrete has a fine structure, although often containing internal cracks. A targeted study on layer PBD shows that 92% of silcrete artefacts from this layer have been heat treated [45], and preliminary observations indicate that silcrete heat treatment is a common behaviour in all layers. Heat treatment has been shown to eliminate internal heterogeneities in silcrete that would have caused breakage of the cores during the knapping procedures. The heating procedure also offered angular fragments with suitable angles to be exploited, which led to fewer constrains on the selection of raw material volumes. The colour variation of silcrete is similar to that observed in the inland “*koppies*” (rocky hills) where silcrete outcrops occur within a 10 km radius around the site. The colour, textural and structural variations of the silcrete types present in the archaeological sample match the variations observed in the reference geological sample collected at several locations close to the site [45]. A waterworn cortical surface on cobbles is most common on cortex-bearing cores and blades (4.5%). This cortex type is sometimes present on waterworn nodules found near the inland silcrete outcrops but this wear may also be due to sea or river abrasion and rolling. Silcrete cobbles have not been found on the beaches near KDS suggesting an inland source is more likely. Calcrete, present in small proportions throughout the sequence, is found at the same source as the silcrete and is also readily available within and around KDS.

### Raw material representation

Fig 2 presents the raw material proportions for each HP layer at KDS, excluding “indeterminate” which represent < 0.2% of the raw material composition of all layers (n = 6, n = 3/6837 respectively). In terms of raw material, there is a strong similarity between the three lower layers PCA, PBE, PBD which show a dominance of silcrete, followed by lesser amounts of quartzite and quartz. In layer PBC, this trend shifts drastically towards a dominance of quartzite, followed by quartz while silcrete only represents 14%. In the overlying layer PBA/PBB, quartz becomes dominant (65%) while the use of silcrete and quartzite is particularly low. The two uppermost layers PAZ and PAY show another change in pattern. From PAZ upwards, quartz decreases, to represent only 13% in PAY while quartzite becomes the main raw material in this layer. Silcrete is better represented than in layers PBC and PBA/PBB, but still remains half as well represented than in the lower layers PCA to PBD. The three uppermost layers PBA/PBB, PAZ and PAY show higher proportions of calcrete and fine quartzite artefacts in comparison to the lower layers. In terms of raw material representation, the sequence can be divided into two main phases: 1) PCA to PBD dominated by silcrete and 2) PBA/PBB and PAZ with a dominance of quartz. Layers PBC and PAY, showing higher amounts of quartzite artefacts seem to stand apart. The techno-typological results below provide further data to refine the phases.

### Knapping techniques and the first steps of core reduction

**Fig 3:**
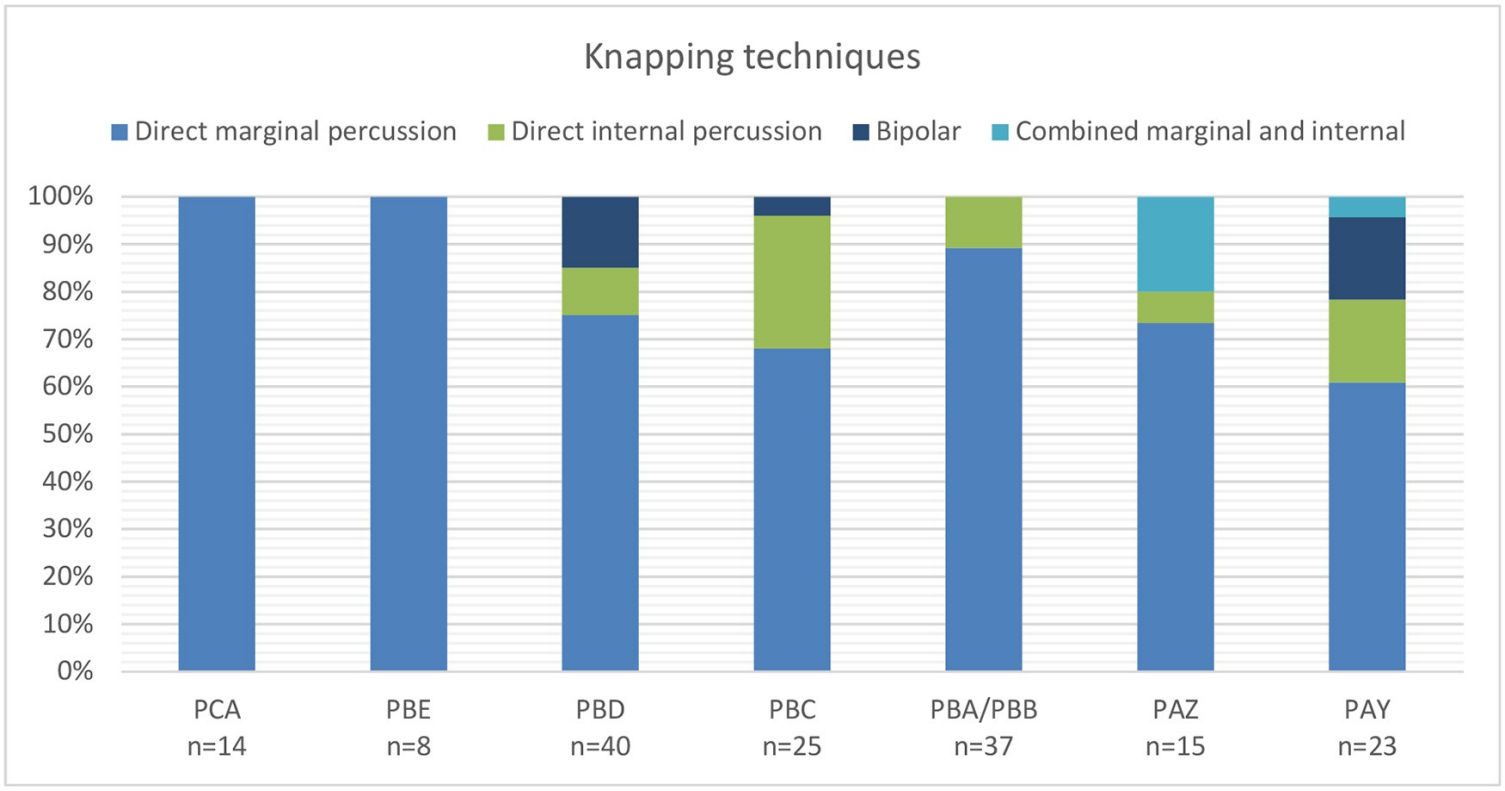
Knapping techniques observed on cores per layer.

The platform characteristics were not recorded systematically on products but the knapping techniques identified on 87% of the cores (n = 177/203) show a strong prevalence for direct marginal percussion throughout the sequence, even for the Levallois core reduction. This technique is identified by weakly developed or absent bulbar scar negatives on cores, indicating a marginal percussion on the knapping platform surface. It is the exclusive technique applied for core exploitation in the two lowest layers, PCA and PBE (Fig 3). In all other layers (PBD to PBA), direct internal percussion, identified on cores by strongly developed negative bulbar scars and identifiable impact areas, is always used in addition to marginal percussion, and in combination with it on the same core in the two upper layers PAZ and PAY.

Bipolar technique on cores, identified by angles close to 90°, rectilinear striking axes and blunting and fissuring, is present in layers PBD, PBC and PAY (Table 2, Fig 3), but identified end-products of bipolar reduction are rare (n = 9/2373 blades; n = 1/4251 flakes) and mainly found in layers PBD and PAY. This discrepancy is probably due to a lack of distinction of bipolar products among the end-products, for example shattered platforms might also result from other knapping techniques.

**Table 2:** Core reduction methods identified in the sequence of KDS.

	PCA		PBE		PBD		PBC		PBA/PBB		PAZ		PAY	
	n	%	n	%	n	%	n	%	n	%	n	%	n	%
Blade/Bladelet unidirectional	3	*19%*	3	*25%*	21	*39%*	5	*17%*	3	*6%*	2	*11%*	-	-
Unidirectional	5	*31%*	3	*25%*	8	*15%*	11	*37%*	10	*21%*	7	*37%*	9	*36%*
Blade/Bladelet bidirectional	2	*13%*	-	-	5	*9%*	2	*7%*	1	*2%*	2	*11%*	4	*16%*
Bipolar	-	-	-	-	6	*11%*	1	*3%*	-	-	-	-	3	*12%*
Discoid	-	-	1	*8%*	6	*11%*	2	*7%*	7	*15%*	-	-	2	*8%*
Recurrent centripetal	-	-	1	*8%*	1	*2%*	1	*3%*	6	*13%*	-	-	-	-
Levallois rec. centripetal	-	-	-	-	-	-	-	-	5	*11%*	1	*5%*	-	-
Levallois rec. unidirectional	-	-	-	-	-	-	1	*3%*	4	*9%*	1	*5%*	5	*20%*
Levallois preferential	-	-	-	-	1	*2%*	-	-	3	*6%*	-	-	1	*4%*
Informal Core	6	*38%*	1	*8%*	5	*9%*	5	*17%*	7	*15%*	4	*21%*	1	*4%*
Core Fragment	-	-	3	*25%*	1	*2%*	2	*7%*	1	*2%*	2	*11%*	-	-
**TOTAL**	**16**	*100*	**12**	*100*	**54**	*100*	**30**	*100*	**47**	*100*	**19**	*100*	**25**	*100*

An abundance of blades and flakes without a cortical surface is observed in all layers, in particular in layers PCA and PBE (Table 3), but in some cases at least, the core reduction started at the site, as indicated by the presence of flakes and blades with more than 50% cortex. From a technological perspective, we additionally identified four silcrete and four quartzite *entame* blades in layer PBD that are not cortical, indicating that the core reduction could also directly start on non-cortical surfaces such as from angular edges that are naturally present on the raw material. This is, for example, supported by the observations made on layer PBD [45], in relation to the heat-treatment of silcrete blocks that produced angular fragments directly suitable for knapping.

**Table 3:** Percentage of cortex on flakes and blades per layer.

	0%	<25%	25–50%	50–75%	>75%	100%	Indet.	Total n =
**PAY**	81.2%	6.9%	3.3%	3.0%	2.5%	2.6%	0.4%	724
**PAZ**	81.1%	7.2%	5.6%	2.4%	2.4%	1.4%	0.0%	502
**PBA/PBB**	75.9%	8.2%	6.8%	4.1%	2.1%	1.8%	1.1%	1496
**PBC**	81.3%	10.4%	3.4%	1.6%	1.3%	1.6%	0.3%	614
**PBD**	73.8%	10.3%	6.5%	3.9%	3.1%	2.1%	0.4%	1780
**PBE**	89.2%	4.6%	1.5%	1.2%	1.9%	1.0%	0.5%	777
**PCA**	93.4%	3.3%	1.2%	1.1%	0.8%	0.1%	0.0%	732

Core management flakes and blades include rare blades with multidirectional dorsal scars. These are mainly found in layer PBA/PBB where centripetal core exploitation is the most frequent reduction method observed (Table 4). *Débordant* blades (n = 81/2373 blades) and *débordant* blades with cortical backs (n = 41/2373 blades) also occur and they represent removals from the lateral edges of unidirectional cores. A few crested blades (n = 14/2373 blades) were also produced during this stage of the preparation of the lateral core convexities.

**Table 4:** Dimensions of the complete blades at KDS expressed in mm.

Layer	N =	Length				Width				Thickness			
		Mean	Max	Min	stdev	Mean	Max	Min	stdev	Mean	Max	Min	stdev
PAY	53	33.49	101.20	12.43	16.97	14.23	53.6	4.4	7.35	6.49	17.5	1.9	4.06
PAZ	24	24.52	40.65	14.3	6.75	11.32	18.24	4.14	4.03	4.36	9.36	2.1	1.65
PBA/PBB	60	27.83	70.41	14.19	10.73	13.30	32.79	5.7	5.15	4.56	11.37	1.8	2.14
PBC	28	28.08	46.4	16.2	8.06	10.7	21.6	5.7	3.43	4.04	12.6	1.3	2.18
PBD	139	27.75	71.2	10.9	9.98	11.88	19.5	4	4.41	3.73	12.6	0.8	2.15
PBE	48	20.27	51.66	8.20	10.51	9.35	22.9	2.8	5.04	3.24	10.3	1	1.94
PCA	46	28.45	66	13.6	12.88	11.29	21.5	4	4.55	3.92	10	1.5	1.74

Discoidal cores occur in relatively higher quantities in layers PBE and PBA/PBB (Table 2). The resulting flakes, including pseudo-Levallois flakes, are absent from the bottom layer PCA and they represent between 0.3 and 1.2% of the flakes in layers PBE to PAZ, with a slightly increased representation of 2.1% in the uppermost layer PAY.

### Core reduction methods and end-products

**Fig 4:**
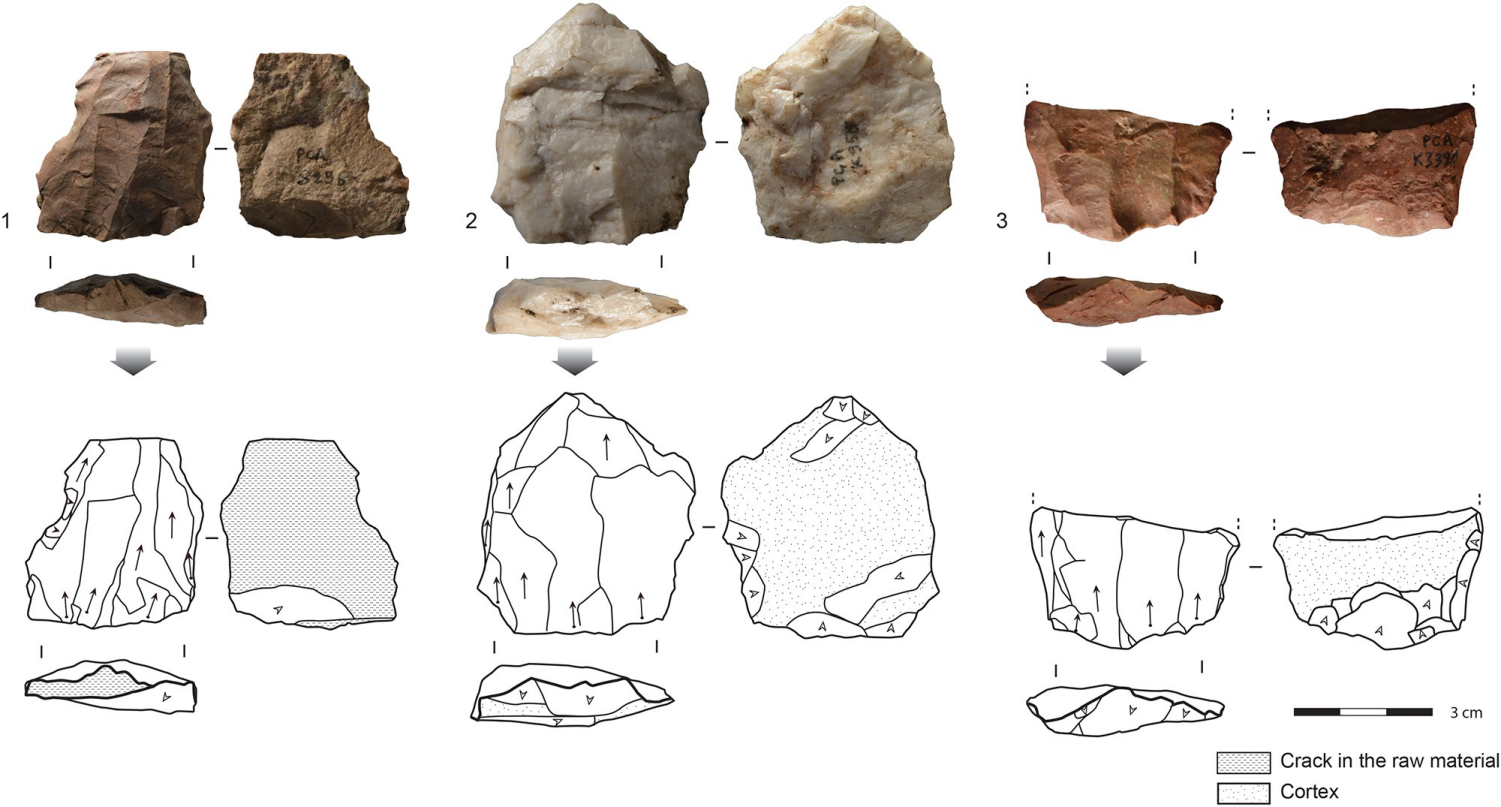
Examples of typical HP cores from layer PCA. 1,3: Silcrete. 2: Quartz.

The majority of the cores in all of the layers show unidirectional exploitation (Table 2). In the lower layers (PCA-PBD), unidirectional exploitation is mainly used for reducing blade cores, whereas in the upper layers (PBC-PAY) unidirectional cores are mostly flake cores. Blade cores include a broad range of core volumetric exploitations, including a small proportions of cores (n = 26/203 cores, 13%) that have initially been described at Klasies River as “Howiesons Poort cores” [36]. They are characterized by a large debitage surface and pronounced lateral convexities. The most typical examples of "HP cores" are found in layer PCA (Fig 4). Unidirectional blade cores produce a broad range of blanks, from narrow blades or bladelets to elongated flakes, most often with dorsal blade scars. Bidirectional exploitations is less frequent, and mostly devoted to the production of blades rather than bidirectional flakes. Bidirectional exploitation show no chronological pattern within the sequence.

The blade production methods and resulting products do not show important technological changes through time. Overall, there is a slight increase in the dimensions of the blades over time and they tend to be more irregular in the uppermost layer PAY (Table 4, [5]).

**Fig 5:**
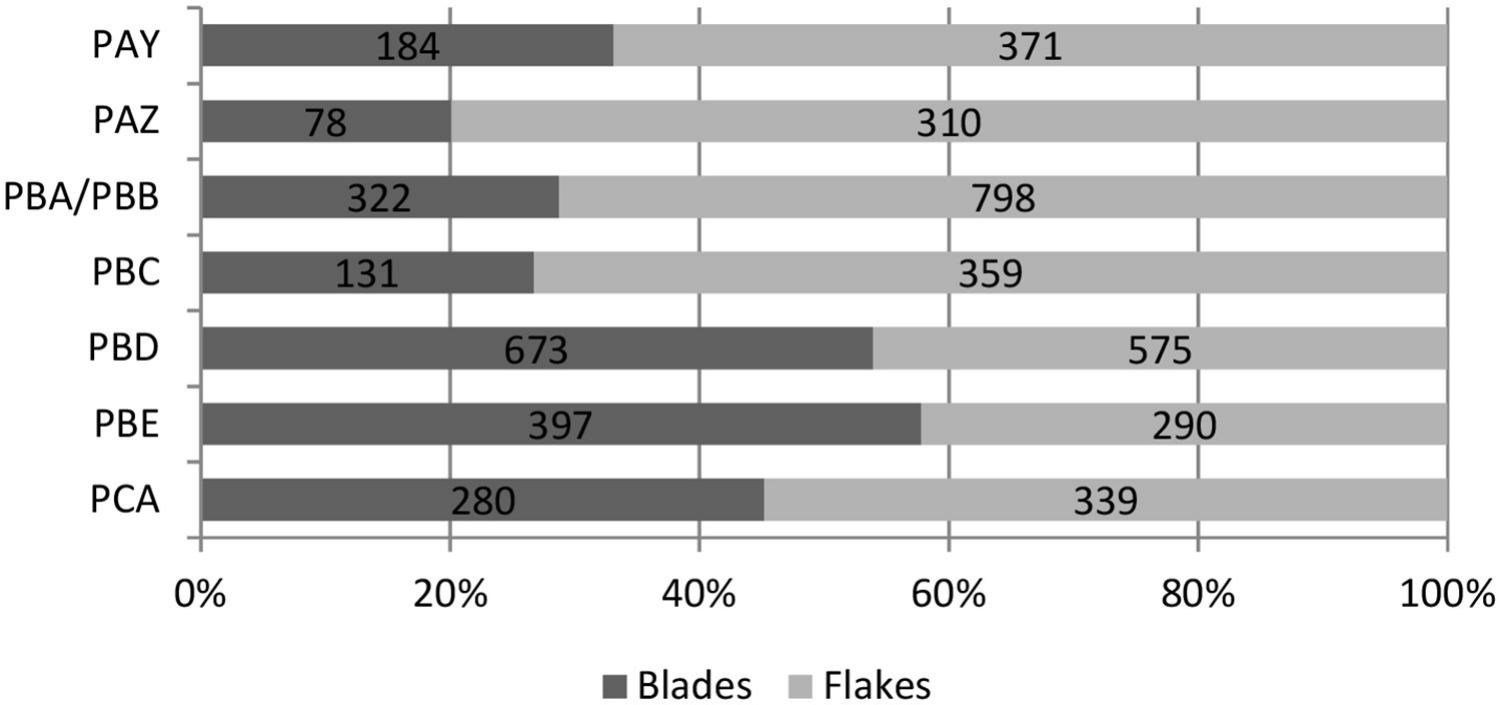
Proportions of blades and flakes in each layer of KDS, excluding core management flakes and blades.

In general, we observe an increase in the production of flakes over time. Flakes in blade-based industries, could most frequently occur as the result of core preparation and core management processes (i.e. *entame*, cortical, *débordant* with and without cortex, convexity preparation products, etc.). At KDS, core management flakes represent normally between 21% and 27% of the flakes of each assemblage, with no noticeable variation through time. The increase in the proportion of flakes over the sequence is not due to a higher degree of core preparation or core management over time, as illustrated in Fig 5. Additionally, the good representation of flakes with dorsal blade scars in the lower part of the sequence (PCA-PBD) shows that flake production is likely integrated in the blade production in these layers, either produced at a late (or last) stage of reduction or occasionally during the reduction. The lack of cores showing formal flake production in these layers supports this interpretation. An independent flake production only emerges from layer PBC onwards, as evidenced by unidirectional, centripetal, discoidal and Levallois cores (Table 2).

**Fig 6:**
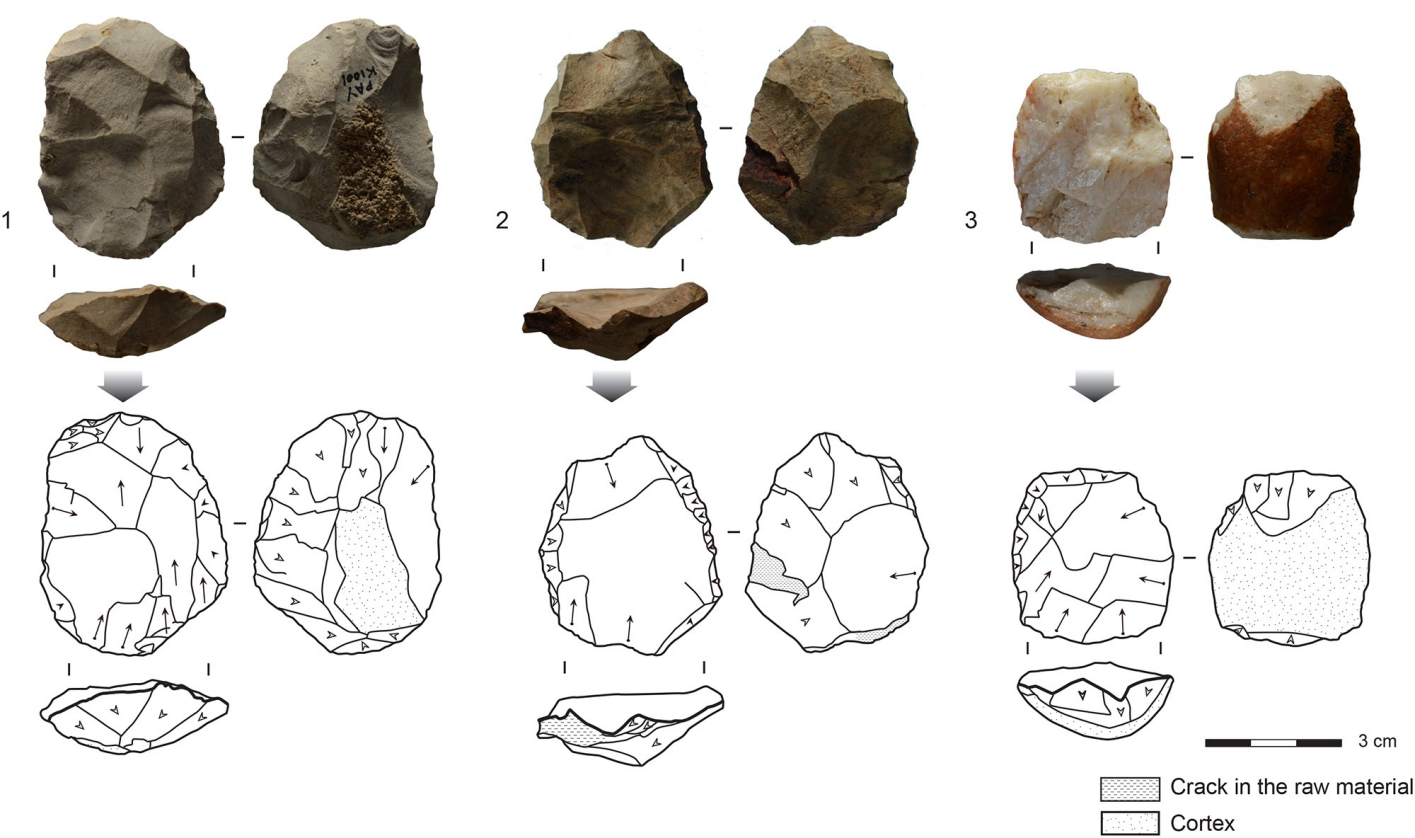
Examples of Levallois cores from the KDS sequence. 1–2: layer PAY, Silcrete. 3: layer PBA/PBB, Quartz.

Cores with a Levallois structure (Fig 6) are well represented, more specifically from layer PBA/PBB upwards. They are characterized by preferential, recurrent unidirectional and recurrent centripetal methods (Table 2). The negative scars of the end-products on these Levallois cores mainly show the production of flakes (n = 10/22) and to a lesser extent elongated flakes (n = 5/22) and blades (n = 6/22). This emergence of an independent flake production is corroborated by the fact that the top layer PAY contains 24 of the 30 typical Levallois flakes identified in the whole sequence; the remaining six being found in layers PAZ (n = 5) and PBA/PBB (n = 1).

### Retouched tool production

There are 176 retouched tools in the whole sequence of KDS, which represent 2 to 3% of the total assemblage for each layer, except for layer PAZ that contains 5% (n = 25/523) of tools. Backed tools comprise the largest tool group of the whole sequence but they do not dominate in each assemblage. They are prevalent in layers PBD to PAZ and they are clearly minor in the oldest layer PCA and in the youngest layer PAY (Table 5).

**Table 5:** Categories of tools identified at KDS.

	PCA		PBE		PBD		PBC		PBA/PBB		PAZ		PAY	
	n	%	n	%	n	%	n	%	n	%	n	%	n	%
**BT**	**3**	**14%**	**5**	**31%**	**13**	**26%**	**9**	**57%**	**20**	**62%**	**9**	**36%**	**1**	**6%**
SCR	1	5%	-	-	3	6%	1	6%	3	9%	3	12%	2	13%
D	-	-	-	-	3	6%	-	-	2	6%	-	-	1	6%
DS	1	5%	-	-	1	2%	-	-	1	3%	-	-	-	-
NSC	1	5%	-	-	-	-	1	6%	2	6%	2	8%	-	-
NSR	5	23%	5	31%	1	2%	1	6%	1	3%	-	-	2	13%
NMR	5	23%	4	25%	1	2%	2	13%	-	-	-	-	-	-
BCR	1	5%	-	-	8	16%	-	-	-	-	-	-	-	-
B	-	-	-	-	5	10%	-	-	-	-	-	-	-	-
PE					4	8%	1	6%	2	6%	1	4%	-	-
R	5	23%	1	6%	9	18%	-	-	-	-	4	16%	4	25%
P	-	-	-	-	-	-	-	-	-	-	-	-	3	19%
BU	-	-	-	-	-	-	1	6%	-	-	2	8%	1	6%
M	-	-	-	-	1	2%	-	-	-	-	3	12%	1	6%
LDR	-	-	1	6%	-	-	-	-	1	3%	1	4%	1	6%
**Total**	**22**	**100%**	**16**	**100%**	**49**	**100%**	**16**	**100%**	**32**	**100%**	**25**	**100%**	**16**	**100%**

BT: backed tools, SCR: scraper, D: denticulate, DS: denticulated scraper, NSC: notched with single clactonian notch, NSR: notched with single retouched notch, NMR: notched with multiple retouched notches, BCR: blade with marginal continuous retouch on one lateral edge, B: borer, PE: *pièce esquillée*, R: undifferentiated retouched, P: point, BU: burin, M: miscellaneous, LDR: localized deep removals. Backed tool types are listed in Table 2.

**Fig 7:**
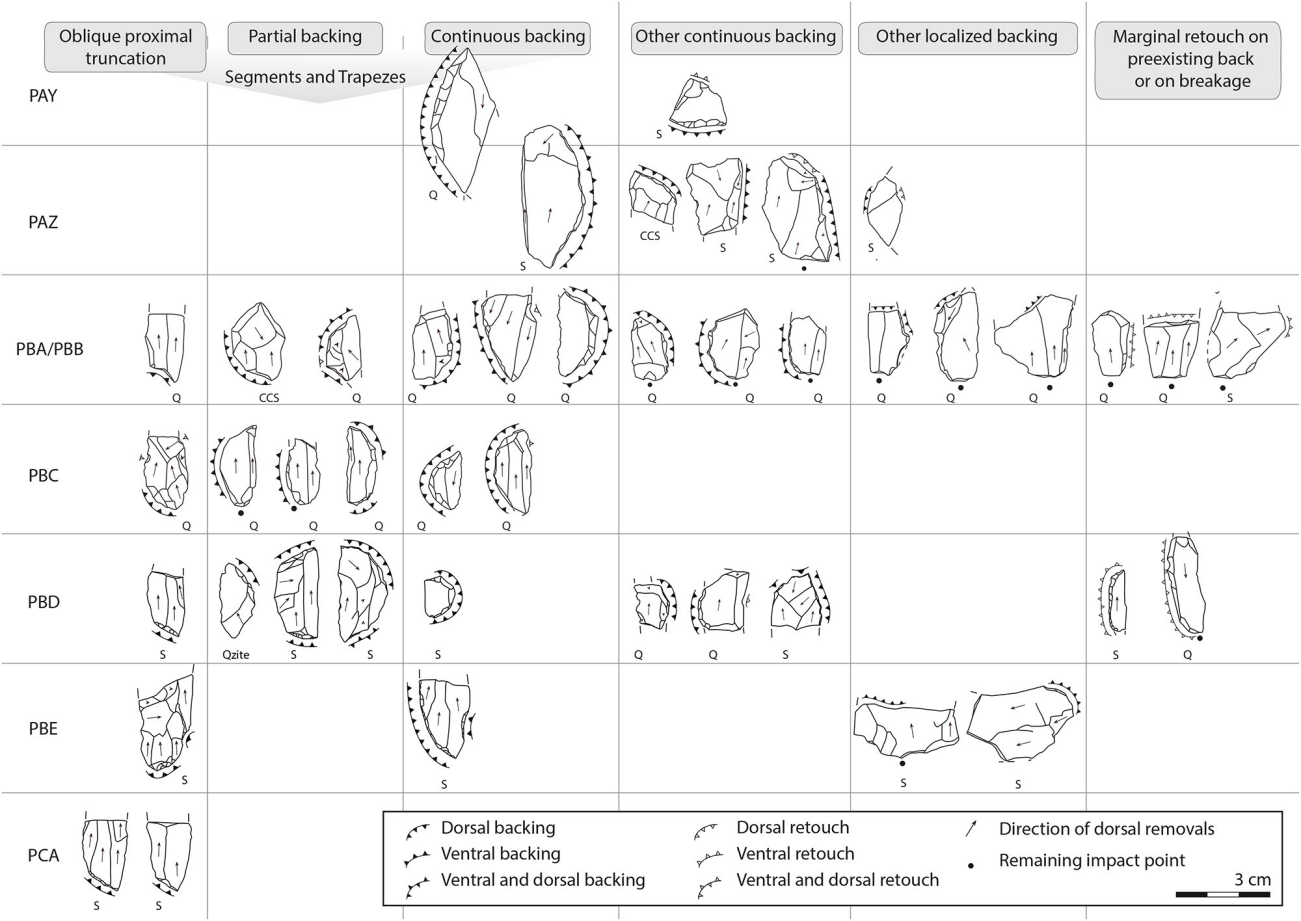
Examples of backed tools identified within the HP sequence of KDS. Raw material is indicated below each tool: S: Silcrete, CCS: Cryptocrystalline silica, Q: Quartz, Qzite: Quartzite. For proportions of each backed tool type per layer, refer to Table 6. Piece with continuous backing illustrated here for layer PAY comes from another ¼ square meter than those studied in this paper. Technical drawings distinguish dorsal from ventral removals as well as backing removals (= abrupt to vertical) from retouch removals that do not create a back and that may occur side by side on a single tool.

We grouped backed tools into six morpho-technical categories (Fig 7), from which some are similar to the categorization established by Deacon [30,46]. These groups take into account 1) the location of the backing in comparison to the overall morphology of the tool blank and/or of the tool morphology, and 2) the continuous or discontinuous presence of backing. We identified:

- Oblique proximal truncation. This refers to a backing process by abrupt retouch that removed the proximal part of the blank obliquely to the striking axis of the blank. The blanks, usually blades, are broken in their distal part.
- Partial backing and continuous backing. This refers to abrupt retouch, either discontinuous or continuous, on the curved edge of the tool, forming a partial or full back, while the opposite cutting edge remains unretouched and relatively straight. The morphologies of partially backed tools are variable, mainly trapezoidal (double opposite oblique backing) and half-moon shaped (segments). Continuously backed tools are usually segments and broken segments.
- Other continuous abrupt backing and localized backing. These categorize tools for which the backing process usually does not result in a segment or a trapezoidal morphology. They are usually made on flakes or irregular blades.
- Marginal retouch on pre-existing back or on breakage. Here the abrupt angle of the back is not formed by backing removals, but by pe-existing backs (generally *débordants*) or breakages that are retouched. These tools can, on occasion, have geometric morphologies like segments and trapezes. This group is similar to what Porraz et al. [19] have named “naturally backed tools”.

The diachronic interpretation of the backed tool proportions through the sequence is made difficult by their low amounts in some layers. However, when considered in association to other tool classes, several trends appear. Tools with oblique proximal truncation seem to be typical of the oldest layers PCA to PBD where they are always made on silcrete blades. These layers are additionally characterized by silcrete strangulated blades and by silcrete blades with a single retouched notch (Table 5). Layers PBE and PBD also contain other backed tool types that are absent from our sample of layer PCA, made equally on blades and flakes (Table 6). Layer PBD stands out in containing regular blades with one continuously retouched edge, the highest proportion of *pièces esquillée*s and the only borers documented in the HP sequence. Borers are made on different types of raw material (2 on silcrete and 3 on milky quartz, crystal quartz and ccs), usually on blades (n = 4/5). Three complete ones (n = 3/5) are between 22 and 34 cm long and show a narrow beak made through marginal retouching, on the distal part of the blank or, in one case, on the proximal end.

**Table 6:** Categories of backed tools identified at KDS.

	PCA n=	PBE n=	PBD n=	PBC n=	PBA/PBB n=	PAZ n=	PAY n=
Continuous backing, geometrics	-	1	1	2	3	2	-
Partial backing, geometrics	-	-	3	3	2	-	-
Oblique proximal truncation	3	1	3	2	1	1	-
Other continuous backing	-	-	4	-	5	4	1
Other localized backing	-	3	-	1	3	1	-
Marginal retouch on pre-existing back or on breakage	-	-	2	1	6	1	-
**Total**	**3**	**5**	**13**	**9**	**20**	**9**	**1**

Total for all layers: n = 60. Geometrics include segments and trapezes.

Segments are typical of layers PBC and PBA/PBB where they are mostly made on quartz (n = 9/10). They are relatively standardized in terms of shape, although their dimensions vary (see S1 Table). In PBC the segments are predominantly made on blades (n = 4/5) while those from PBA/PBB are made on flakes (n = 4/5). In the other layers (PBE, PBD, PAZ and PAY), segments are made on various raw materials and they are less standardized in shape.

**Fig 8:**
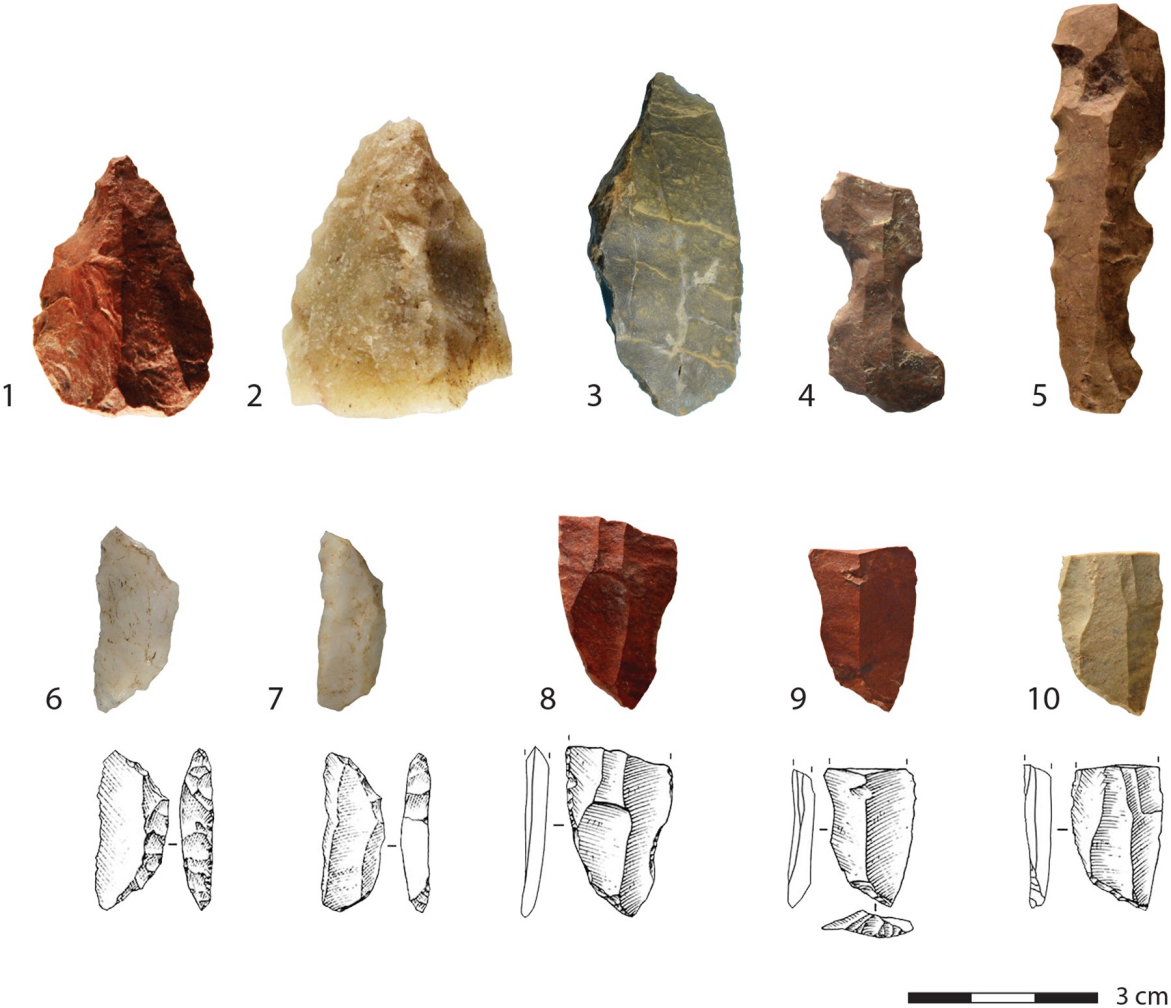
Examples of tools from the HP sequence of KDS. 1–2: PAY; 3: PAZ; 4,8: PBE; 5,7: PBC; 6: PBA/PBB; 9–10: PCA. 1,3,4,5,8,9,10: Silcrete; 2: Quartzite; 6–7: Quartzite. Drawings by Gauthier Devilder after [5].

Layers PAZ and PAY, at the top of the sequence show an increased number of scrapers and miscellaneous tools (Table 5). Additionally, in the uppermost layer PAY points made on flakes appear, two in silcrete and one in coarse quartzite. One is complete (Fig 8: 1) and shows dorsal and ventral covering retouch, while the two other pieces only show dorsal retouch.

It is notable that six silcrete cores have been recycled into scrapers or miscellaneous tools (PBD: n = 3; PBE, PAZ, PAY: n = 1 respectively).

## Discussion

The lithics within the HP are well known for their range of technological features such as the production of blade/bladelets by marginal percussion, the presence of backed tools, the selection of fine-grained raw material and heating of silcrete (e.g. [14,15,17,45]) that make this period unique within the MSA. The disappearance of these features at the onset of the post-HP is the subject of extensive discussion including scenarios based on demography [39,40,47,48,49] and others that consider technological change as an adaptive response to changing environmental conditions and mobility strategies [49, 50,51]. A few studies [5,19,35,36,37,52–55] have also highlighted patterns of changes within HP sequences although long HP records across southern Africa are rare. Among these, the KDS lithic sequence allows us to assess the mechanisms of technological changes that are particularly relevant to understand the evolutionary dynamics within the HP, but also in relation to the post-HP transition.

### Patterns of technological change at Klipdrift Shelter

**Fig 9:**
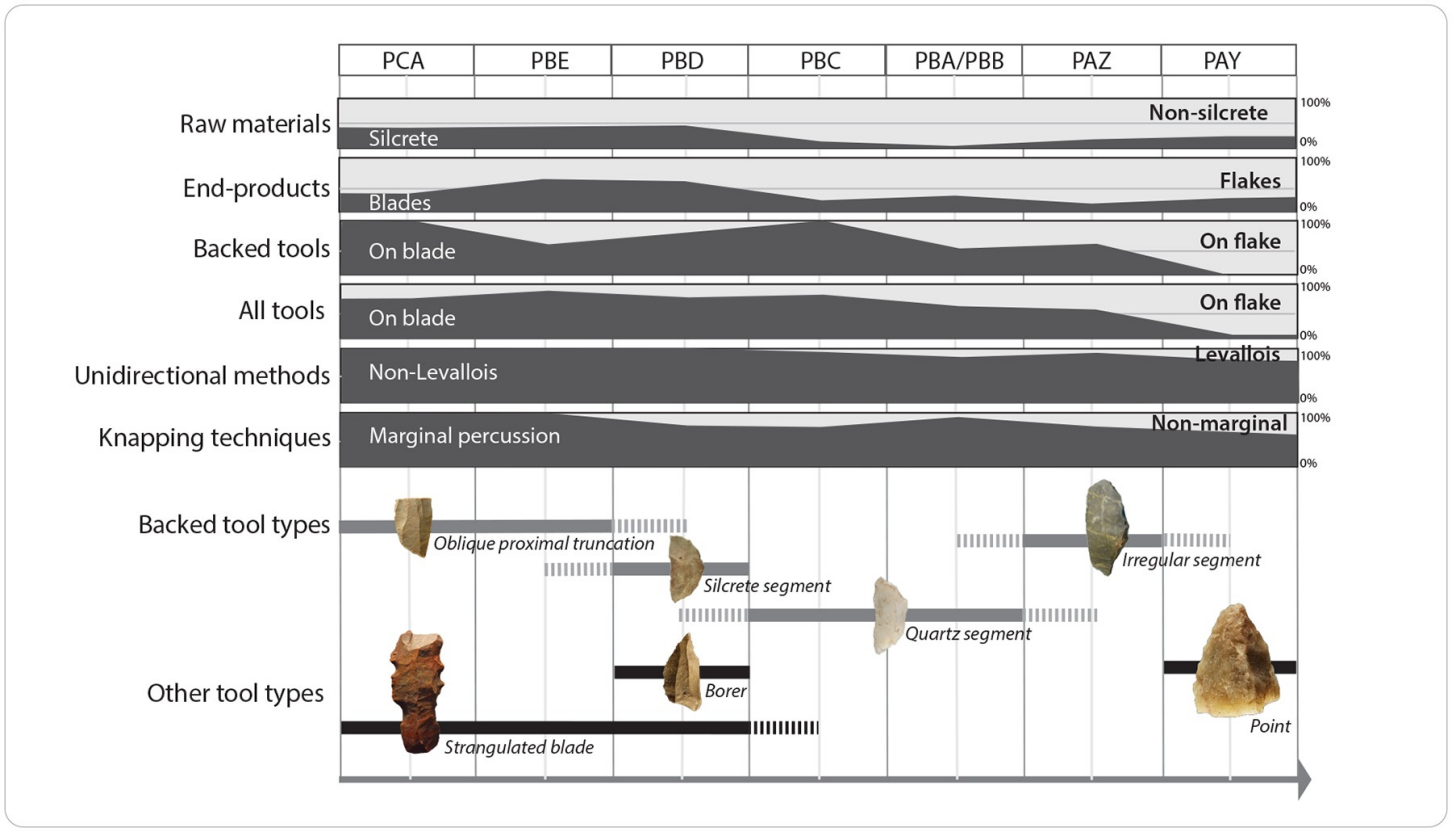
Pattern of techno-typological changes in lithic assemblages through the HP sequence at KDS.

At KDS, the lithic assemblages show a number of stable features within the sequence, expressed in a basic technological package which essentially remains stable throughout the sequence and that is typical of the Howiesons Poort industries in South Africa. It includes the persistence of marginal percussion as a main knapping technique, the dominance of unidirectional core reduction methods in all layers, the persistence of blade production as well as the manufacture of backed tools. On the other hand, varied patterns of changes are observed (Fig 9), including abrupt shifts, gradual shifts and time-restricted shifts, depending on the proxy.

At KDS, drastic shifts are observed in raw material selection and blank production. Raw material selection is marked by an abrupt shift after layer PBD, when silcrete is replaced as dominant raw material in layers PCA to PBD, by quartzite in PBC and PAY and by quartz in PBA/PBB. Concomitant abrupt changes are visible in the proportions of blades that decrease drastically in the upper layers (PBC to PAY), as flake production increases significantly. The correlation between these two abrupt shifts can be seen as the result of the strong preference for silcrete for blade production, especially since the intentional procedure of silcrete heating is greatly facilitating blade production [45]. There is however a certain time lag before seeing the effects of these shifts on tool manufacturing. This is best expressed in layer PBC, where the proportions of silcrete and blades drop while blades remain the most frequently selected blanks for tool manufacturing, and for the proportions of backed tools that continue to increase. It is only from layer PBA/PBB upwards that a more significant selection of flakes as blanks for tool manufacturing is present, including use for backed tools. Layer PBC thus provides evidence of a transitional stage, expressed by an abrupt techno-economic turnover observed in raw material selection and blank production together with a certain continuity in tool manufacturing.

These abrupt shifts are accompanied by more gradual changes through time. The gradual shift from blades to flakes as dominant blank types for tool manufacturing, is accompanied by the development of Levallois production methods, increased proportions of wider blades and an overall decrease in their morphological standardization.

Successive time-restricted shifts occur in the tools’ typology. Backed tools show noticeable changes in their typology, although they are present in all layers. Proximal oblique truncations on silcrete blades are the most outstanding backed tools found in layer PCA and PBD, replaced by quartz segments in PBC and PBA/PBB, while irregular segments prevail in the upper layer PAZ and presumably in PAY, although only one example is recorded for this layer. A difference is backing intensity exists between obliquely truncated silcrete blades and quartz segments, but it seems probable that these two categories of backed tools are the result of different tool conceptions rather than the result of different stages of reduction induced by raw material selection. The typological variations evidenced for this typical HP tool category, i.e. backed tools, can neither be directly related to changing blank production strategies, as they are equally produced on flakes or blades (S1 Table). Their typological variations could reflect changing hafting techniques, a hypothesis which needs to be tested through functional analyses. Similar time-restricted occurrences apply to tool types with specific morphofunctional characteristics, such as strangulated blades (mainly in PCA to PBD), borers (PBD) and points (PAY). The high variability in tool type composition other than backed tools over time, cannot be directly connected with any abrupt or gradual technological shifts, as pointed out before, and can thus rather be seen as the expression of changing activities performed by the HP groups, either at the site scale or at the more limited scale of the excavated area.

Changes in lithic strategies at KDS are complex, represented by asynchronous and different temporalities as well as by cumulative patterns, but they also provide an opportunity to compare the phasing of the HP sequence at KDS with changes observed in other HP sequences across southern Africa.

### A sequence from the Intermediate HP to the post-HP

Other well documented HP lithic sequences include Rose Cottage dated between 66±4ka to 59±4ka [35] and Sibudu PGS layer dated to 64.7±1.9ka and GR layer dated to 61.7±1.5ka [17,56], located in the north-eastern part of South Africa. KDS which is dated between 65.5±4.8 ka and 59.4±4.6 ka [5] also compares with sites from the south western part of South Africa such as Klein Kliphuis dated to 66±3 ka and 65 ± 3 ka [41], Klasies River main site dated to ca. 65 ka [57] and Diepkloof Rockshelter. Geochronological analysis of the sequence of Diepkloof Rock Shelter indicate an early HP dated from 109±10 ka to 85±9 ka, an intermediate HP dated from 85±9 ka to 65±8 ka, and of a late HP dated from 65±8 ka to 52±5ka [18–19]. The occurrence of an early HP phase at Diepkloof Rock Shelter has led the authors to argue that “The record of Diepkloof supports an early appearance of the HP in the Western Cape followed by a later diffusion across the rest of southern Africa. » ([37] p.3550). Apart from the early HP documented only at Diepkloof, there is a general concordance between all southern African HP sequences in terms of chronology [16], and also in terms of pattern of changes, although a certain degree of regional variability occurs.

The lower part of the KDS sequence (PCA to PBD) can be compared to the “classic HP” as identified at Rose Cottage Cave (layers EMD to MAS), characterized by careful blade production with very marginal percussion, core reduction sequences for blade production and backed pieces, and an almost exclusive use of opaline as fine-grained raw material which is not found at KDS [35]. At Rose Cottage Cave and Sibudu (Layers GR, GS and PGS) backed tools are represented in much larger proportions than at KDS [17]. However, Layers PCA to PBD at KDS even better corresponds with the definition of the “intermediate HP” as described for Diepkloof Rockshelter (stratigraphic units Joy to Fred) that is also characterized by the overall preference for fine-grained silcrete and for strangulated-notched pieces while backed elements form a minor component of the toolkits [19]. Strangulated blades are also present in the HP sequences of Klasies River [1] and Klein Kliphuis [41]. The use of silcrete heat-treatment for blade production, as evidenced in layer PBD [45] is found in other HP assemblages in south-western South Africa, all characterized by a predominance of silcrete. This behavior is documented in units Frank and Frans at Diepkloof, which correspond to the upper layers of the “intermediate HP” [58], in Mertenhof from “lower HP” to “post-HP” layers [59] and at Pinnacle Point site 5–6 for aggregate DBCS attributed to the HP dating between 65 ± 3 ka and 60 ± 2 ka [60,61].

Layers PBA/PBB and PAZ at KDS show a relatively higher amount of naturally backed elements with marginal subsequent retouch, a pattern that can be compared to the production of naturally backed flakes at the top of the HP sequence at Diepkoof Rockshelter [19]. This “late HP” sequence (stratigraphic units Frans to Debbie) as recognized at Diepkloof, contains more silcrete than at KDS where quartz is dominant, although there is an increasing proportion of quartz between sub-phases Frans and Eric at Diepkloof. This PBA/PBB-PAZ phase also resembles the changes observed at Klasies River where there is an increase in flake production that starts in the Middle HP and continues in the Upper HP with a possible independent flake production strategy that is recognized as early as the Lower HP [36]. Additionally, at Klasies River, as for KDS, quartz raw material selection becomes more frequent in square E50, layers CP18-CP12, the lower part of the Upper HP phase, and a similar pattern occurs at Klein Kliphuis from the 7 upper spits of Dvi upwards [41]. Finding an explanation for the abandonment of silcrete use in favour of quartz and quartzite at the onset of the Late HP is still elusive, in particular as it has been suggested by some that raw material selection (i.e. silcrete) is not influenced by environmental or climatic conditions, or sea level changes [13,23] but see [49,55,62]. At Klasies River, it has been suggested that the drop of fine silcrete in favour of quartz and quartzite could be due to “a decline in productivity of known supply sources” ([12]:636). We emphasize that this gradual abandonment could be linked with a lesser need for thin narrow blades in fine grained raw material such as silcrete (see also [41]), the production of which also requires heat treatment [45]. The sequence of Sibudu [17,56], is more difficult to correlate to KDS, although it rather resembles this later KDS phase than the bottom “intermediate HP” phase. At Sibudu, a clear flake component is also recognized as part of the result of multiple coexisting core-reduction methods in the Grey Rocky Layer [56]. In the Sibudu sequence backed tools are dominant but there is a decline in their frequency through time. At Rose Cottage Cave the representation of side-scrapers increases in the “final HP” phase (layers ETH to SUZ) [35] as is the case at KDS.

The PAY phase at KDS contains transitional characteristics but it could possibly represent a first phase of the post-HP. Unfortunately, the layers above PAY did not provide enough material to better define the post-HP at KDS. In some respects this assemblage shows strong similarities with the post-HP at Klein Kliphuis [41] and at Diepkloof Rockshelter [19,37]. At Klein Kliphuis, as at KDS, there is no clear discontinuity between the HP and the post-HP, but a shift in raw material and the co-existence of a few backed tools with the appearance of unifacial points. At Diepkloof Rockshelter, the transition from the HP to the post-HP is not accompanied by any change in raw material, but shows a progressive change with the manufacture of scrapers and the increased importance of flake production. This pattern of gradual change is also present at KDS with an increasing selection of flakes as tool blanks and an intensification of independent flake production by Levallois methods from layer PBC to PAY. Layer PAY therefore also complies with the definition of the post-HP sequence at Rose Cottage [35] where Levallois flakes are more important and secondary raw materials, such as volcanic tuff at Rose Cottage (or calcrete in layer PAY at KDS), are more frequently used. The attribution of PAY to the “final HP” or to the post-HP is made difficult in the context of the gradual nature of this transition. However, it clearly shows a more gradual process than at Sibudu, where a drastic change in tool classes between the upper HP and the post HP is recognized, leading us to interpret this transition as a rapid disappearance of the HP [17]. In contrast to sites from the north-east of southern Africa (e.g. Sibudu, Rose Cottage Cave, Umhlatuzana, etc.) where uni-bifacial points occur in different stages of the HP [22,23], the presence of unifacial points in the south-western region could be a valid typological marker for the emergence of the Post-HP as they do not appear in the intermediate or late HP in this region ([21]; but for early HP see [19]).

It appears that at KDS, as in other HP sequences in southern Africa, a fundamental technological shift occurs between the intermediate HP and Late HP with the initiation of a new technical system based on flake production. This transition is well documented at KDS in layer PBC. While its impact on the typological characteristics within the later assemblages occurs at a different pace, this transition is an early indication of the gradual emergence of the post-HP. The main driving force put forward to explain the reorganisation of the technical systems between the HP and the Post- HP, is the change in environmental conditions and its effect on resource availability and group mobility induced by a gradual aridification that started at the end of MIS 4 and becomes more pronounced during MIS 3 (e.g. [42,51,53]). This supposes that there could be a co-evolution between environmental conditions, subsistence strategies, lithic technical strategies and possibly also with symbolic behaviors. While the comparison of faunal data and subsistence strategies available for different southern African HP sites is difficult to interpret [51], KDS offers a range of anthropogenic remains to interpret the patterns of changes and potential driving forces on a refined scale.

### Behavioral versus environmental (a)synchronies

When searching for mechanism of cultural changes, environmental data are significant on a local scale insofar they affect faunal and mineral resource availability and group mobility, due to variations in vegetation cover and sea levels (e.g. [42,43,55]). If synchronous changes in proxies occur, it may be possible to suggest a causal relationship, either demographic or environmental. Asynchronous patterns of changes between different proxies may rather indicate more complex dynamics, less prone to be influenced by a single catastrophic triggering event (ie., demographic collapse; climatic variations; population replacement), such as sustained and interactive inter- and intra-group contact or the introduction and maintenance of highly innovative behaviors.

A few basic components are stable through the HP sequence. In terms of site function, KDS was likely a living site where roasting, skinning, filleting, dismembering and marrow extraction activities are relevant to all layers [42]. Shellfish are collected across the various phases in the KDS sequence; there is an extensive use of a variety of ochre types for different activities, and the use and/or consumption of ostrich eggs is common.

**Fig 10:**
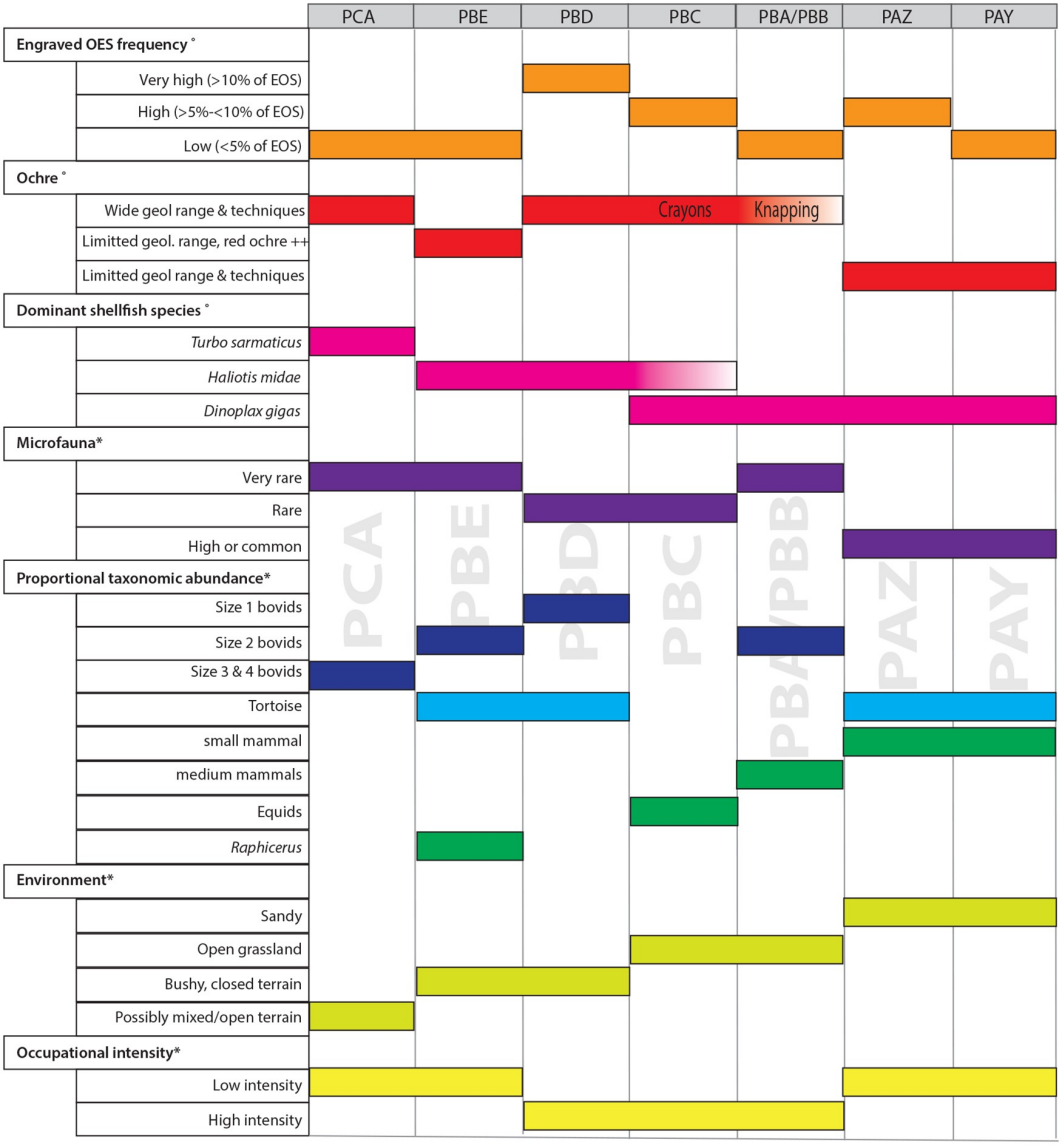
Synthetic representation of pattern of changes in non-lithic assemblage characteristics through the sequence. Proxies *: adapted from [43]. Proxies °: adapted from [5].

A finer scale of analysis reveals that in the lower part of KDS sequence (layers PCA to PBD), a number of proxies show important changes [43] (Fig 10) in contrast to the stable features observed for the lithic assemblages. The transition from PCA to PBE in particular shows a shift from possibly mixed or open terrain to bushy or closed vegetation, a reduction in the size of bovids, a significant presence of *Raphicerus*, the introduction of tortoise in the diet and a shift in shellfish dominant species from *Turbo sarmaticus* to *Haliotis midae* [5]. The ochre in layer PBE stands out within the sequence with a dominant selection of red ochres originating from a limited geological range. Also, the engraved OES frequency rises considerably from below 5% of OES to 12.4% of OES between layer PBE and layer PBD [5]. This accumulation of changes has not led to any significant shift in lithic raw material acquisition and lithic technological behaviors at KDS.

The transitional period featured in the lithic record from layer PBC is correlated with a change from bushy, closed terrain to open grasslands, expressed by a shift from bovid representation towards equids between layers PBD and PBC, as well as a strong decrease in tortoise taxa. A significant increase in *Dinoplax gigas* is also observed.

Between PBA/PBB and PAZ, all environmental and behavioral proxies evidence a major shift, with the exception of the lithic record which remains stable. The environment changes from open grassland to sandy terrain, and the faunal remains show important increases in the relative amounts of tortoise and small mammals. Ochre exploitation between PBA/PBB and PAZ shifts towards much more limited geological ranges as well as to limited technical uses [5]. By contrast, in the uppermost layer PAY, the lithics evidence a shift towards point production and a significant increase in Levallois flake production while all other proxies, whether environmental or related to subsistence strategies remain stable between layers PAZ and PAY.

When comparing patterns of change in the lithics with that in the other proxies we identify diametrically opposed trends. When the lithic strategies are stable (PCA to PBD and PBA/PBB to PAZ) the other proxies show changes. On the contrary, the most prominent changes in the lithic record, such as those recorded between layers PAZ and PAY with the emergence of points and the increase use of Levallois methods, take place during a stable phase with regards to all the other proxies. Ultimately, only the abrupt changes evidenced from PBC, ie. between the Intermediate to Late HP, co-occur with other major changes, in particular with a marked opening up of the environment. This leads to the conclusion that at KDS, with the exception of PBC, changes in lithic strategies are generally not synchronous with changes in subsistence behaviors or environmental conditions.

One additional proxy, the engraved OES, requires further analysis to be fully applicable for a comprehensive diachronic study, but it provides indications of changes that are specific to PAZ and PAY. The exhaustive study of the engraved designs is ongoing but preliminary results seem to show that one specific motif, the “finely carved diamond shaped cross-hatched pattern” [5:295], only appears in the HP sequence from PAZ to PAY and is the exclusive motif of engraved OES in PAY. In layers PBC to PAZ, the sub-parallel rectilinear or curved lines motif would be the most common. Further analyses are needed to confirm the exclusivity of this design to the two upper layers, but it seem to accompany the general behavioral change towards the Post-HP. Interestingly, the extensive study of engraved OES at Diepkloof [6, 63] has shown that, besides synchronic variability, designs show a drastic shift between the Intermediate HP (Governor to Fred) and the Late HP (Edgar to Debbie). While orthogonal hatched and obliquely hatched bands are dominant in the Intermediate HP, convergent or sub-parallel striations and the increase in motifs by intersection of two series of hatching occur dominantly in the Late HP, before engravings disappear in the Post-HP. According to these results, there is a close relationship between changes in lithic strategies and in the behaviors that are related to the use of these OES.

The dynamic changes observed at KDS are unlikely caused by dramatic events such as population replacements. The overall similarities of the HP successions on a regional scale but also on the wider regional scale rule out the assumption that behavioural changes at KDS are solely driven by local innovations. Strong arguments in favour of interactions with other HP populations are even better supported by the occurrence of engraved ostrich eggshells both at KDS [5] and Diepkloof Rockshelter [6,63] but also farther north, at the Namibian site Apollo 11, in HP layers dated to 63.2±1.9 ka [57,64]. Engraved ostrich eggshells from these three sites share similar designs of engravings that feature varied cross-hatched and sub-parallel line themes. However, the “sub-parallel intersecting lines motif” recognized at Apollo 11 and that dominates in Late HP layers at Diepkloof, is absent at KDS so far. In turn, the finely carved diamond shaped cross-hatched pattern mentioned above for KDS, is absent at Diepkloof and Apollo 11. Texier and colleagues [63] who produced a comprehensive study of MSA engraved OES, have demonstrated their use as containers and they suggest in [37] that engravings are symbols that were used to mediate intragroup social interactions, in a period of extended social networks that would have reached its climax during the classic HP [37]. Consequently, a set of designs could have constituted a common symbolic ground for the HP groups but with unique variants occurring in different localities, lithic technological HP repertoires share very close patterns on a large scale but with regional variations. For example, the presence of strangulated blades for the south-western HP sites and point productions for the north-eastern sites, led Mackay et al. ([21]:44) to suggest that “these differences reflect continuing regional traditions over-printed by similarities arising from interactions”. The regional variability in HP cultural behaviours may reflect different adaptations to the variable environmental responses to climatic changes induced by the aridification at the end of MIS 4 in the three main rainfall zones (south western—winter rainfall, southern Cape—year round rainfall, northeast coast and interior, summer rainfall). At KDS, with the exception of layer PBC, there is no clear co-occurrence of changes in lithic strategies and environmental conditions. While faunal spectrums and resource availability may be highly dependent on environmental changes, leading to a rapid adaptation of subsistence strategies [43], the behavioural response of lithic production strategies may have evolved on a broader temporal scale (see also [65]). The emergence of a dominant flake technology in layer PBC at KDS has an irreversible impact on the HP techno-economical system and marks the initiation of the post-HP. The coincidence of the drastic turnover in lithic strategies in this layer with significant environmental change characterized by an opening of the vegetation, has yet to be understood and considered carefully. But it could represent the only argument, if any, supporting a rapid adaptation to changing environmental conditions which could have triggered the emergence of new subsistence strategies, which may have resulted in the replacement of backed tools by pointed implements that presumably do not have the same function (eg. [51]).

## Conclusion

We regard the internal variability observed at KDS and at other HP sites within southern Africa as the expression of a highly inventive and dynamic culture, open to changes and driven by strong social interactions, especially at a regional scale. This leads to strong recurrences in the patterns of changes during the HP over large spatial scales. At KDS the changes in lithic strategies are generally not synchronous with changes in symbolic behaviours, subsistence behaviours or environmental conditions—with the exception of layer PBC. This opens avenues for discussion about the relevance of models of behavioural evolution during the HP based on single proxies. Layer PBC at KDS marks the most profound shift that sees the gradual introduction of components that are typical of the post-HP. The transition to the post-HP, and the effective termination of HP technologies, starts with the shift from the Intermediate to the Late HP well before the appearance of the typical post-HP unifacial points. This transitional process provides a marker for the disappearance of the HP from the southern African MSA.

## Supporting information

S1 Table Dimensions of backed tools per type and raw material (expressed in mm). Blanks: FL: Flake, BL: Blade, Indet.: Indeterminate.
(DOCX)
