# Supplementary material for: The Howiesons Poort lithic sequence of Klipdrift Shelter, southern Cape, South Africa
Source: PLoS One. 2018 Nov 7;13(11):e0206238. doi: 10.1371/journal.pone.0206238 (PMC6221302; doi:10.1371/journal.pone.0206238)
Supplement: S1 Table — Blanks: FL: Flake, BL: Blade, Indet.: Indeterminate. (DOCX) [file pone.0206238.s001.docx]

Supporting Information:

S1 Table: Dimensions of backed tools per type and raw material (expressed in mm). Blanks: FL: Flake, BL: Blade, Indet.: Indeterminate.

| **Layer** | **Type** | **Raw material** | **Break** | **Length** | **Width** | **Thickness** | **Platform** | **Blank** |
| --- | --- | --- | --- | --- | --- | --- | --- | --- |
| PBD | Backed, continuous retouch | Silcrete | ? |  | 11.70 | 3.30 |  | FL |
| PBD | Backed, continuous retouch | Silcrete | Distal |  | 20.05 | 3.98 |  | FL |
| PBD | Backed, continuous retouch | Milky quartz | Mesial |  | 14.30 | 4.32 |  | BL |
| PBD | Backed, continuous retouch | Milky quartz | Distal |  | 28.20 | 5.20 |  | FL |
| PBA/PBB | Backed, continuous retouch | Milky quartz | Complete | 28.56 | 21.98 | 5.27 | Facetted | FL |
| PBA/PBB | Backed, continuous retouch | Milky quartz | Complete | 27.49 | 15.68 | 4.99 | Indet. | FL |
| PBA/PBB | Backed, continuous retouch | Milky quartz | Complete | 45.29 | 22.27 | 6.76 | Facetted | BL |
| PBA/PBB | Backed, continuous retouch | Milky quartz | Proximal |  | 15.53 | 2.22 | Very small | FL |
| PBA/PBB | Backed, continuous retouch | Milky quartz | Complete | 24.4 | 15.86 | 5.11 | Facetted | FL |
| PAZ | Backed, continuous retouch | Silcrete | Complete | 43.53 | 25.7 | 8.95 | Facetted | FL |
| PAZ | Backed, continuous retouch | Milky quartz | Proximal |  | 22.46 | 6.55 | Retouched | FL |
| PAZ | Backed, continuous retouch | Silcrete | Distal |  | 22.94 | 4.54 |  | BL |
| PAZ | Backed, continuous retouch | Chert | Distal |  | 16.71 | 3.86 |  | Indet. |
| PAY | Backed, continuous retouch | Silcrete | Distal |  | 19.36 | 6.22 |  | FL |
| PBE | Backed, localized retouch | Silcrete | Distal |  | 10.81 | 3.05 |  | BL |
| PBE | Backed, localized retouch | Silcrete | ? | 20.92 | 33.9 | 7.47 |  | FL |
| PBE | Backed, localized retouch | Silcrete | Proximal |  | 33.55 | 6.56 | Facetted | FL |
| PBC | Backed, localized retouch | Silcrete | Complete | 34.8 | 10.7 | 4.8 | Very small | BL |
| PBA/PBB | Backed, localized retouch | Milky quartz | Complete | 21.79 | 13.63 | 3.49 | Indet. | BL |
| PBA/PBB | Backed, localized retouch | Milky quartz | Complete | 30.15 | 14.75 | 5.15 | Dihedral | BL |
| PBA/PBB | Backed, localized retouch | Milky quartz | Complete | 34.18 | 17.35 | 3.95 | Facetted | BL |
| PAZ | Backed, localized retouch | Silcrete | ? | 23.57 | 12.46 | 2.8 |  | FL |
| PBD | Backed, marginal retouch | Milky quartz | Complete | 31.22 | 11.38 | 5.02 | Facetted | BL |
| PBD | Backed, marginal retouch | Silcrete | Mesial |  | 5.71 | 3.61 |  | BL |
| PBC | Backed, marginal retouch | Milky quartz | Distal |  | 10.79 | 3.31 |  | BL |
| PBA/PBB | Backed, marginal retouch | Milky quartz | Complete | 26.19 | 10.87 | 4.25 | Shattered | BL |
| PBA/PBB | Backed, marginal retouch | Milky quartz | Complete | 19.22 | 18.34 | 4.63 | Facetted | BL |
| PBA/PBB | Backed, marginal retouch | Milky quartz | Mesio-distal |  | 12.39 | 3.74 |  | BL |
| PBA/PBB | Backed, marginal retouch | Silcrete | Proximal |  | 24.84 | 5.03 | Facetted | FL |
| PBA/PBB | Backed, marginal retouch | Milky quartz | Proximal |  | 12.4 | 6.18 | Facetted | BL |
| PBA/PBB | Backed, marginal retouch | Milky quartz | Mesial |  | 14.55 | 5.39 |  | FL |
| PAZ | Backed, marginal retouch | Milky quartz | Mesial |  | 15.93 | 3.76 |  | BL |
| PCA | Oblique truncation | Silcrete | Proximal | 23.11 | 14.71 | 2.38 | Retouched | BL |
| PCA | Oblique truncation | Silcrete | Proximal | 22.33 | 14.68 | 3.49 | Retouched | BL |
| PCA | Oblique truncation | Silcrete | Proximal | 21.66 | 15.24 | 4.45 | Retouched | BL |
| PBE | Oblique truncation | Silcrete | Proximal |  | 17.4 | 3.17 | Retouched | BL |
| PBD | Oblique truncation | Silcrete | Distal |  | 11.18 | 2.85 |  | BL |
| PBD | Oblique truncation | Silcrete | ? |  | 17.4 | 4 |  | FL |
| PBD | Oblique truncation | Silcrete | Proximal |  | 11.90 | 2.40 | Retouched | BL |
| PBC | Oblique truncation | Milky quartz | Proximal |  | 7.7 | 1.8 | Retouched | BL |
| PBC | Oblique truncation | Silcrete | Complete | 38 | 16.1 | 6 | Shattered | BL |
| PBA/PBB | Oblique truncation | Milky quartz | Proximal | 23.21 | 12.85 | 4.1 | Retouched | BL |
| PAZ | Oblique truncation | Milky quartz | Complete | 40.65 | 17.81 | 9.36 | Retouched | BL |
| PBE | Geometric, complete backing | Silcrete | Proximal |  | 16.94 | 3.3 | Retouched | BL |
| PBD | Geometric, complete backing | Silcrete | Complete | 13.78 | 10.78 | 4.96 | Retouched | FL |
| PBC | Geometric, complete backing | Milky quartz | Complete | 19.18 | 10.25 | 3.66 | Retouched | FL |
| PBC | Geometric, complete backing | Milky quartz | Complete | 27.55 | 12.49 | 3.45 | Retouched | BL |
| PBA/PBB | Geometric, complete backing | Milky quartz | Complete | 23.81 | 12.95 | 5.02 | Retouched | FL |
| PBA/PBB | Geometric, complete backing | Milky quartz | Complete | 28.36 | 13.13 | 5.24 | Retouched | FL |
| PBA/PBB | Geometric, complete backing | Milky quartz | Proximal |  | 18.13 | 5.93 | Retouched | BL |
| PAZ | Geometric, complete backing | Milky quartz | Distal |  | 14.5 | 5.9 |  | BL |
| PAZ | Geometric, complete backing | Silcrete | Complete | 47.61 | 21.41 | 6.31 | Very small | BL |
| PBD | Geometric, partial backing | Quartzite | Complete | 24.54 | 11.23 | 4.78 | Retouched | FL |
| PBD | Geometric, partial backing | Milky quartz | Complete | 24.4 | 11.6 | 2.8 | Plain | BL |
| PBD | Geometric, partial backing | Silcrete | Complete | 31.37 | 16.64 | 4.13 | Retouched | BL |
| PBC | Geometric, partial backing | Milky quartz | Complete | 21.17 | 11.13 | 2.5 | Very small | BL |
| PBC | Geometric, partial backing | Milky quartz | Complete | 26.64 | 11.63 | 2.38 | Very small | BL |
| PBC | Geometric, partial backing | Milky quartz | Complete | 25.9 | 9.52 | 3.92 | Retouched | BL |
| PBA/PBB | Geometric, partial backing | Milky quartz | Complete | 22.29 | 11.57 | 5.35 | Retouched | FL |
| PBA/PBB | Geometric, partial backing | Chert | Complete | 25.79 | 17.86 | 3.2 | Retouched | FL |
